# Inhibitory control deficits and ventral attention network alterations linked to apathy in schizophrenia

**DOI:** 10.1038/s41398-026-04066-7

**Published:** 2026-05-21

**Authors:** Wenrui Deng, Marie-José van Tol, Jan-Bernard C. Marsman, Claire Kos, Michelle N. Servaas, André Aleman

**Affiliations:** 1 Center for Clinical Neuroscience and Cognition, University Medical Center Groningen, University of Groningen, Groningen, the Netherlands; 2 Research School of Behavioural and Cognitive Neurosciences, University of Groningen, Groningen, the Netherlands; 3 Clinical Cognitive Neuro-Psychiatry research program (CCNP), Department of Psychiatry, University Medical Center Groningen, Groningen, the Netherlands; 4 Faculty of Psychology and Neuroscience, Maastricht University, Maastricht, the Netherlands

**Keywords:** Schizophrenia, Human behaviour

## Abstract

Individuals with schizophrenia demonstrated impaired inhibitory control and apathy symptoms, which are characterized by a reduction in self-initiated voluntary activities. However, whether deficits in inhibitory control are linked to apathy remains unclear. Here we investigated inhibitory control-related neural networks and associations with interview-based apathy and actigraphy-derived motor activity, an objective measure of apathy. Twenty-three patients with schizophrenia underwent 3T-fMRI scans during Go/No-Go task. Task-related networks were identified via independent component analysis. Behavioral performance was examined using a drift diffusion model. Correlation and regression analyses examined relationships between apathy severity scores, motor activity, behavioral parameters, task-related networks engagement and connectivity. Patients with higher levels of apathy showed reduced motor activity levels, lower overall accuracy rate in inhibitory control, particularly more inhibitory errors, lower Go-condition processing efficiency, and reduced stimulus sensitivity. On the neural level, higher apathy correlated with weaker ventral attention network (vAN) engagement during successful inhibition, and weaker default mode network (DMN) engagement during failed inhibition, although the latter association was influenced by age and depressive symptoms. Stronger left posterior cingulate cortex spatial contribution to DMN and weaker vAN-DMN coupling were associated with higher apathy in patients with schizophrenia. Motor activity levels were unrelated to inhibitory-related networks. Higher apathy in schizophrenia was related to worse inhibitory control performance and decreased vAN and DMN recruitment and connectivity during response inhibition, indicating impairments in sustaining attention on the task and allocating cognitive resources in response to external demands. Dysfunctional neural networks underpinning inhibition may contribute to reduced goal-directed behavior. Motor activity appears to be related to apathy in terms of auto-activation, but not in terms of cognitive inhibitory control.

## Introduction

Apathy is a prevalent and debilitating symptom in neurodegenerative disorders, traumatic brain injury, and schizophrenia [1]. It is characterized by a reduction in self-initiated voluntary activities and goal-directed behaviors, manifesting as deficits in cognitive, emotional, and auto-activation dimensions [2]. In schizophrenia, apathy is a core feature of negative symptoms, particularly including difficulties with the initiation and maintenance of behaviors necessary to achieve goals [3]. Over 50% of individuals at high-risk for psychosis and after a first-episode of psychosis exhibit apathy and motivational impairments, which tend to worsen with the progression of the disease [4, 5]. High levels of apathy have been shown to correlate with poorer occupational and social functioning [6], as well as lower quality of life [7]. Despite the high prevalence of apathy across various conditions, its neurocognitive mechanisms remain unclear.

Apathy is associated with impaired executive functioning in schizophrenia [8, 9]. Neural mechanisms underlying executive functioning, specifically planning and cognitive set-shifting, have also been linked to apathy in schizophrenia [10] and non-clinical populations [11]. Inhibitory control, a key executive function, involves the ability to suppress undesirable actions that conflict with goal-directed behavior [12]. This process is critical for self-regulation and goal achievement, and is commonly assessed using Go/No-Go response inhibition tasks that require withholding a prepotent motor response [13]. Disruptions in inhibitory control may relate to impaired ability to suppress the effects of interference and distractor, or to conduct self-voluntary activity in a specific context, contributing to apathy. However, whether the neural basis underlying inhibitory control is related to apathy in patients with schizophrenia has not yet been investigated.

In neuroimaging studies using a Go/No-Go task, neural activity during successful inhibition (i.e., correctly withholding a response to No-Go stimuli) and inhibition errors (i.e., false alarm responses to No-Go stimuli) is compared to neural activity during prepotent Go responses (i.e., correct responses to Go stimuli) [14]. The frontoparietal network (FPN), including the right inferior frontal gyrus (rIFG), anterior cingulate cortex (ACC), supplementary motor area (SMA), inferior parietal lobules (IPL), is consistently reported to be involved in successful inhibition in the Go/No-Go task in healthy individuals [13, 15]. Furthermore, subcortical regions such as the amygdala and basal ganglia, along with temporal areas (e.g., the middle temporal gyrus), also contribute to the response inhibition [16–18]. Inhibition errors engage the medial prefrontal cortex, ACC, and insula in healthy volunteers [19, 20]. Several of these regions are part of the ventral attention network (vAN; e.g., superior temporal sulcus, IPL and IFG), which has been implied in inhibitory control [21]. Altered recruitment of the FPN and vAN may disrupt inhibitory control processes, such as stimulus discrimination, action withholding, and cancellation, resulting in inhibition errors [16, 22]. Notably, recent study reported that patients with schizophrenia exhibited altered DMN involvement during Go/No-Go task compared to healthy controls [23]. Taken together, the FPN, vAN, and DMN are implicated in inhibitory control and may therefore be relevant for reductions in goal-directed behavior related to apathy. Moreover, dysfunctions in the ventral striatum, ACC and IPL within a fronto-parieto-striatal network have been associated with apathy across neuropsychiatric disorders [24–26]. Thus, these shared neural networks may relate to apathy in the context of inhibitory control during the Go/No-Go task in patients with schizophrenia.

The performance of inhibitory control is evaluated with reaction time and accuracy. Additionally, the drift diffusion model (DDM) enhances the decoding of inhibitory control by modeling response time and response accuracy to quantify latent cognitive processes based on evidence accumulation framework for decision [20, 27, 28]. This provides information on individual differences in terms of cognitive processes underlying inhibition. For example, the drift rate (*v*) reflects the speed of evidence accumulation to make a motor response or withhold action decision, modulated by alertness and deficit of attention [27, 29]. This approach allows us to investigate whether apathy modulates specific cognitive processes, and their neural substrates.

Reduced motor activity (i.e., lower quantity and less variability of motor activity as measured by actigraphy) correlates with apathy assessed by clinical interviews in patients with schizophrenia, and is suggested to be an objective measurement for apathy [30, 31]. Furthermore, motor activity and clinical apathy measures show divergent associations with neural activations in the IFG and ventral striatum during reward anticipation [30], suggesting that motor activity may capture a certain aspect of apathy. To examine whether reductions in motor activity are related to inhibitory control deficits, we investigate their relationships.

The present study aimed to investigate associations between apathy severity, neural networks and behavioral parameters during inhibitory control in patients with schizophrenia and high levels of apathy. Apathy was measured by Apathy Evaluation Scale (AES), and motor activity parameters derived from actigraphy served as objective indicators of apathy. We expected that higher levels of apathy would correlate with lower motor activity. Given that patients with schizophrenia showed to have impairments in cognitive control, we hypothesized that higher levels of apathy or lower motor activity were correlated with a higher false alarm rate, and reduced sensitivity to Go stimuli in the inhibition task. Because the common underlying regions of apathy and inhibition are in the FPN, vAN and DMN, we expected that higher levels of apathy or lower motor activity were associated with FPN, vAN, and DMN engagement during successful inhibition. Finally, we explored the functional network connectivity related to apathy.

## Materials and methods

### Participants

Twenty-three patients diagnosed by a clinical psychiatrist with schizophrenia according to DSM-IV criteria and exhibiting apathy were included in this study. Apathy was assessed using the clinical version of Apathy Evaluation Scale (AES-C) administered by trained research assistants, with a cutoff score of 27 on the 12-item AES-apathy subscale [32, 33]. This cutoff score is based on a prior study of two standard deviations above the mean of AES-apathy in a healthy reference group, indicating clinical apathy [4]. The presence of diagnosis was confirmed using the Mini-International Neuropsychiatric Interview (M.I.N.I.-Plus) [34]. Further inclusion criteria were aged between 18 and 65 years, and on stable antipsychotic medication use for at least four weeks. Exclusion criteria included (1) diagnoses of neurological disorders or head injury; (2) a current diagnosis of substance dependence disorders; (3) any condition incompatible with magnetic resonance imaging (MRI), such as claustrophobia, metal implants, or pregnancy. The data of this study were part of the baseline measurements from a treatment trial aimed at improving apathy, approved by the Medical Ethics Committee of the University Medical Center Groningen (trialregister.nl: NTR3805). Informed consent was obtained from all participants and this research was conducted in compliance with the declaration of Helsinki.

### Clinical outcome measures

The Positive and Negative Syndrome Scale (PANSS) was used to measure positive, negative, and general psychopathology [35]. The Scale for Assessment of Negative Symptoms (SANS) measured negative symptoms across five domains: affective flattening, emotional blunting/alogia, avolition/apathy, anhedonia/asociality, and attentional impairment [36]. Depressive symptoms were assessed by using the Calgary Depression Scale for Schizophrenia (CDSS), designed to measure depression independently of negative symptoms of schizophrenia [37]. The Temporal Experience of Pleasure Scale (TEPS) was used to evaluate anhedonia including both the anticipatory and consummatory experiences of pleasure [38].

### Actigraphy

Participants wore an actigraphy device (ActiCal, Respironics, Inc., Murrysville, PA) around their non-dominate wrist over two consecutive weekend days following clinical assessments. Actigraphy was measured with one-minute intervals. Weekends were selected because exercise activities during this time are less affected by external factors, such as work, therapy, or school, leading to more self-initiated participation [39–41]. For further details on data recording see Servaas et al. (2019). We analyzed the levels and variability of activity during the most active ten hours per day to minimize the inclusion of rest periods. The *activity levels* were calculated as the average activity counts per minute for each participant. The root of mean squared successive difference (RMSSD) was used to measure *activity variability* based on successive changes, because it well depicts both variability and temporal dependency in a time series [42, 43]. One participant was excluded from the motor activity analyses due to exceptionally high *activity levels* exceeding four standard deviations above the mean activity counts of all participants.

### Go/No-Go task

Participants performed a well-researched Go/No-Go task during fMRI scanning to assess inhibitory control (Hershey et al., 2004). Two types of stimuli required different responses, pressing the button when a Go stimulus (non-X letters, e.g., A, B, C) was presented, or withholding a response when a No-Go stimulus (the letter X) occurred. Participants were instructed to press the button as quickly as possible for Go stimuli. The task consisted of 12 blocks with 24 stimuli per block, for a total of 288 trials. Twenty percent of these trials were No-Go. Each stimulus was displayed for 450 ms with an interval of 1000 ms. Performance indexes included the overall accuracy rate (proportion of correct trials), hit rate (proportion of correct Go responses in Go trials), false alarm rate (proportion of false alarm responses in No-Go trials), response time of hits and false-alarms and response sensitivity of signal detection d prime (i.e., d′ = z(hit%) – z(FA%). A higher d′ reflects better perceptual discrimination of Go stimuli versus No-Go stimuli. Data with an overall accuracy rate above 0.50 were included in the analysis, indicating that participants were engaged in the task, did not fall asleep, and the accuracy was more than randomly guessing. Three participants were excluded because they did not meet this accuracy.

Only trials with reaction times over 200 ms were included, as those faster are likely guesses [44]. After excluding these fast reaction trials, all participants had over 200 trials available for model fitting. A basic drift diffusion model fits the response time and accuracy of trials. The model parameters included the drift rate (*v*), representing evidence accumulation rate and performance efficiency, separately for Go and No-Go conditions; the starting point (*z*), indicating response bias; the decision boundary (*a*); and the non-decision time (*ter*) (see supplementary Table S1 for details).

### fMRI Image processing procedure

#### fMRI preprocessing

Details on fMRI acquisition parameters are available in the supplementary materials. The fMRI data was preprocessed using SPM12 (v7771) implemented in Matlab (R2021b; MathWorks) following standard procedures. Functional images were realigned, then co-registered to the structural image, spatially normalized to the standardized space, and smoothed using an 8*8*8 mm full width at half maximum (FWHM) Gaussian kernel.

#### Independent component analysis

Group ICA of fMRI Toolbox (GIFTv4.0b; http://icatb.sourceforge.net/) was used to generate independent components (ICs) with distinct time courses (Calhoun et al., 2008). The preprocessed images were initially reduced using principal component analysis (PCA) and the number of ICs was estimated using the minimum description length (MDL) criteria. The Infomax algorithm was applied to decompose ICs across participants with ICASSO used to generate 20 ICA iterations to determine the reliability and stability of the ICA algorithm. Individual spatial maps were back reconstructed with calculated weights in each voxel, reflecting the strength of functional connectivity between the time courses of each voxel within the component and the average time courses of this component. Functional networks were identified against artifactual components by visually inspecting spatial maps and power spectra, using the following criteria: (1) predominant activity primarily within gray matter, (2) a high low-frequency to high-frequency power ratio in the power spectra distribution, and (3) the time courses dominated by low frequency fluctuations (Cordes et al., 2000). To identify functional networks most similar to brain networks of interest, spatial sorting function in the GIFT toolbox was performed using multiple linear regressions on the spatial maps of generated ICs and the common large-scale brain networks [45, 46].

#### Task-related modulation

The generalized linear model was applied to individual time courses to generate a design matrix for specific task conditions using SPM12. Separate regressors for the onsets and durations of hits (correct Go trials), correct No-Go, false alarms, and incorrect Go trials were included, along with six realignment parameters for head movement and a constant term. To examine the task relevance of each component, multiple regressions between the time courses of network and canonical hemodynamic response model of task were analyzed with the temporal sorting function in GIFT toolbox. The yielded beta weights reflected network engagement in each conditions (i.e., positive or negative betas indicated an increased or decreased in network activity for a particular task condition relative to the baseline) [47]. One-sample t-tests were performed with the stats on beta weights function, to determine whether the beta weights for each task condition in each network was significantly different from zero (i.e., the network is not engaging in the task condition related activity) [48]. Because the hit, false alarm, and No-Go correct were the regressors of interest, components with beta weights that significantly differed from zero in one of these three conditions were identified as task-relevant networks.

#### Within- and between- network connectivity

To examine associations between apathy and within network connectivity (i.e., the spatial contribution within the network) across the task, we first performed one-sample t-tests in SPM12 were based on the spatial map of task-related networks of each participant with voxel-wise family-wise error (FWE) correction (*p*_FWE_ < 0.05) as a threshold to identify regions significantly involved in the component [19, 49]. Because individual component maps include voxels with weak intensity determined by artifacts, which may lead to false-positive results. The multiple regression analyses were performed with AES total scores, and motor activity as covariates, respectively, and restricted to regions that significantly contributed to task-related networks [50]. Multiple comparisons were corrected using the false discovery rate (FDR) method (*p*_FDR_ < 0.05) with an initial uncorrected voxel-wise height-threshold of *p* <0.005. To assess between-network connectivity, time courses of task-relevant networks for each subject were detrended, despiked, and filtered using the MANOVAN function in GIFT. The average time course of the network was calculated by averaging the time courses of all voxels within the network. Functional network connectivity (FNC) was calculated as the Pearson correlation r between paired networks across the task for each participant, then standardized using Fisher’s z-transformation.

### Associations between brain networks and performance of inhibitory control with apathy and motor activity

First, associations between the performance of Go/No-Go task and 1) apathy (AES total score) and 2) motor activity variables (i.e., *activity levels* and *activity variability*) were tested using Spearman correlations. Results were considered significant at *p* < 0.05, two-tailed (uncorrected for multiple comparisons). Second, beta weights of task-relevant networks on task condition contrasts (i.e., No-Go correct > hit, FA > hit) were examined. Associations between the engagement of task-relevant networks for success inhibition or error inhibition and (1) apathy and (2) motor activity variables were tested using Spearman correlations. Results were considered significant at *p* < 0.05, two-tailed (uncorrected for multiple comparisons). Third, the associations of apathy and motor activity with the connectivity within- and between- network were tested by adding the AES total scores, *activity levels* or *activity variability* as a covariate in regression analyses. Because network connectivity involved multiple comparisons across numerous voxel connections, substantially increasing the risk of Type I error. Cluster-wise FDR correction was applied to these analyses to control for multiple comparisons. Associations between ROI-based activations of Go/No-Go task and apathy or motor activity were provided in supplementary materials.

## Results

### Demographic and clinical characteristics

A total of twenty-three patients with schizophrenia participated in this study. Demographic and clinical characteristics of participants were presented in Table 1. Participants showed high levels of apathy (mean = 47.7, SD = 5.21, the distribution of AES presents in supplemental Fig. S1), and AES total scores were positively correlated with age (r = 0.49, *p* = 0.016) and the age of onset (r = 0.43, *p* = 0.039). Apathy, as measured by AES total scores, was not associated with depression (CDSS) (r = 0.30, *p* = 0.188) or anhedonia (TEPS) (r = −0.20, *p* = 0.202).

**Table 1:** Demographic and clinical characteristics of participants (N = 23), Go/No-Go task and weekend motor activity (N = 20). and relations to apathy and motor activity.

	Mean	SD	min	max	median	r _(AES total)_	*p*	r _(Activity levels)_	*p*	*r*_*(Activity variability)*_	*p*
**Demographic and clinical characteristics**											
Sex (female; %)	5 (21.7%)					-		-		-	
Handedness (left)	3 (13%)					-		-		-	
Diagnosis (SZ/SZ-A)	17/6					-		-		-	
Age	31.52	7.46	19	49	29	0.49	0.016	−0.33	0.161	−0.36	0.121
Year of education	16	2.95	12	22	16	0.05	0.817	0.14	0.562	0.23	0.335
Psychoses episodes	3.13	2.51	1	10	2	−0.15	0.491	0.02	0.933	−0.07	0.767
Age of onset	21.74	5.82	11	37	22	0.43	0.039	−0.12	0.624	−0.04	0.851
Duration illness	9.13	5.64	2	25	8	−0.01	0.969	−0.2	0.387	−0.26	0.275
AES Total	47.70	5.21	39	58	47	-		−0.5	0.024	−0.43	0.055
AES Core apathy^a^	29.43	2.45	25	33	30	0.83	<0.001	−0.60	0.006	−0.41	0.075
AES Selected apathy^a^	34.83	2.79	29	40	35	0.86	<0.001	−0.62	0.004	−0.45	0.044
PANSS Total	67.43	12.89	49	95	63	0.73	<0.001	−0.52	0.019	−0.53	0.017
PANSS Positive	13.74	4.38	7	23	14	0.45	0.029	−0.23	0.331	−0.34	0.144
PANSS Negative	18.70	3.44	13	24	18	0.47	0.024	−0.68	<0.001	−0.69	<0.001
PANSS General	35.00	7.59	25	49	34	0.73	<0.001	−0.41	0.073	−0.32	0.170
PANSS Apathy^b^	9.70	2.70	5	15	9	0.50	0.015	−0.6	0.005	−0.71	<0.001
PANSS Expressive deficits^b^	14.78	3.58	9	21	14	0.37	0.081	−0.49	0.027	−0.46	0.043
SANS Total	58.35	9.75	38	78	60	0.58	0.004	−0.82	<0.001	−0.70	<0.001
SANS affective blunting	18.83	6.77	9	32	18	−0.13	0.548	−0.49	0.029	−0.54	0.013
SANS alogia	8.35	2.99	4	17	8	0.23	0.290	−0.21	0.368	0.11	0.631
SANS avolition - apathy	15.22	2.71	10	20	15	0.59	0.003	−0.25	0.293	−0.10	0.666
SANS anhedonia - asociality	15.96	4.29	5	21	17	0.80	<0.001	−0.57	0.009	−0.56	0.011
CDSS^c^	4.19	2.822	0	9	5	0.30	0.188	−0.38	0.121	−0.18	0.475
TEPS Total^c^	64.11	14.69	32	89	65.5	−0.20	0.424	0.52	0.04	0.28	0.300
TEPS consummatory	29.44	9.12	11	44	35	−0.14	0.572	0.5	0.047	0.31	0.237
TEPS anticipatory	34.67	7.65	15	49	30.5	−0.32	0.202	0.41	0.118	0.19	0.488
Activity levels (counts/ min)^c^	292.83	152.41	15.11	578.93	297.60	−0.50	0.024	-	-	0.82	<0.001
Activity variability (counts/min)^c^	361.70	128.72	70.78	611.68	368.49	−0.43	0.055	0.82	<0.001	-	-
**Go/No-Go task - Performance**											
Overall accuracy rate [%]	77	11	53	9	79	−0.55	0.011	0.03	0.911	0.31	0.233
Hit rate [%]	80	15	43	96	85	−0.24	0.300	−0.10	0.708	0.24	0.355
False Alarm rate [%]	36	23	3	86	31	0.41	0.073	−0.21	0.422	0.07	0.783
Hit RT (ms)	330.43	32.37	271.12	381.66	327.04	0.01	0.967	−0.01	0.955	−0.30	0.236
False Alarm RT (ms)	317.68	32.69	249.96	374.5	318.7	−0.08	0.740	−0.12	0.660	−0.36	0.158
d prime	0	1.03	−1.77	1.49	0.03	−0.65	0.0018	0.09	0.722	0.24	0.363
**Go/No-Go task – Drift diffusion model**											
*v*._go_	2.44	0.89	1.19	3.78	2.4	−0.61	0.0046	0.12	0.653	0.24	0.348
*a*	0.59	0.07	0.42	0.7	0.61	−0.04	0.872	−0.13	0.613	0.29	0.260
*ter*	0.27	0.04	0.21	0.34	0.26	−0.09	0.706	0.03	0.896	−0.31	0.232
*z*	0.32	0.09	0.14	0.46	0.33	0.16	0.498	−0.24	0.348	0.14	0.599
*v*._nogo_	−1.74	1.23	−4.29	1.45	−1.78	0.41	0.074	−0.23	0.374	0.03	0.918

^a^AES core score was the sum of ten items, measuring the common core features of apathy in different neuropsychiatric samples [72]. AES selected apathy score was the sum of twelve items measuring the apathy symptoms [33].

^b^Apathy/social amotivation and expressive deficits were two subdomains of negative symptoms from PANSS [73].

^c^Two records of CDSS scores were missing. Five records of TEPS scores were missing. One participant was excluded from motor activity analysis.

Correlations with apathy and motor activity were not corrected for multiple comparisons.

*AES* the apathy evaluation scale, *PANSS* the positive and negative syndrome scale, *SANS* the scale for the assessment of negative symptoms, *CDSS* the calgary depression scale for schizophrenia patients, *TEPS* the temporal experience of pleasure scale, *v*._*go*_ drift rate for go condition, *a* decision threshold, *ter* non-decision time, *z* starting-point bias, *v*._*nogo*_ drift rate for no-go condition.

### Motor activity

**Fig. 1:**
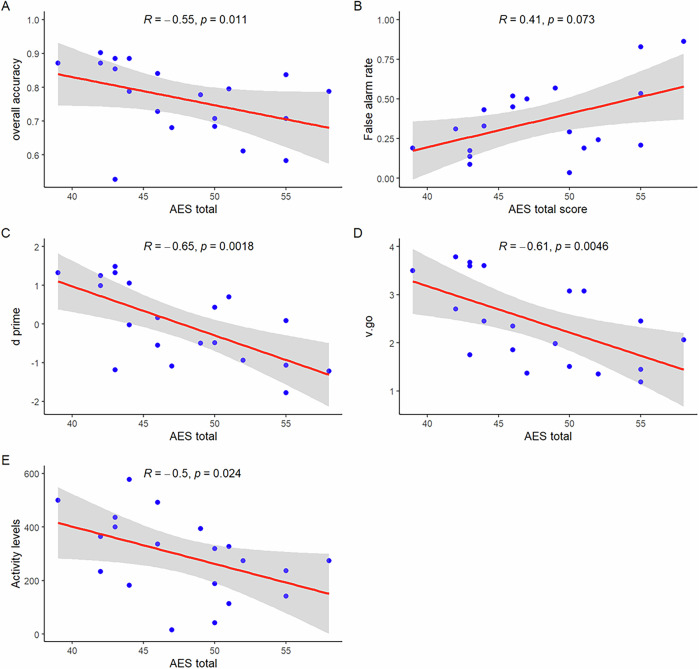
The relationship between the severity of apathy with behavioral task performance and motor activity measures. **A** Negative association between AES total score and overall accuracy. **B** Positive association between AES total score and false alarm rate. **C** Negative association between AES total score and d prime. **D** Negative association between AES total score and *v.*_*go*_ parameter value. **E** Negative association between AES total score and activity levels. Blue dots represent individual participants. Correlation coefficients and corresponding *p* values are shown in each panel.

*Activity level* during the most active 10 h (M10) was 292.83 (SD = 152.41) counts/min, and *activity variability* (i.e., RMSSD) during the M10 time window was 361.7 (SD = 128.72) counts/min (see Table 1). The AES total scores were shown to be significantly negatively correlated with *activity levels* (r = −0.50, *p* = 0.024, Fig. 1D), and have a marginally significant negative correlation with the variability of activity (r = −0.43, *p* = 0.055). No significant relationship was observed between Go/No-Go task (performance and model parameters) and *activity levels*.

### Go/No-Go task

Twenty patients reached an overall accuracy above 50% on the Go/No-Go task, with detailed task performance indexes outlined in Table 1. On average, participants showed an FA rate of 36%, and a hit rate of 80%. Participants responded faster on trials where they failed to withhold their response to No-Go stimuli. A paired t-test comparing response time (RT) for hits and false alarms showed a faster RT for false alarms than for hits (t = 3.06, *p* = 0.006), suggesting a speed-accuracy trade-off. The inhibitory control processes were further decoded using drift diffusion modelling, and results are shown in Table 1.

Spearman correlations revealed that AES total scores were negatively correlated with overall task accuracy (r = −0.55, *p* = 0.011, Fig. 1A) and showed a positive tendency with the FA rate (r = 0.41, *p* = 0.073). Sensitivity of stimulus (d prime) was negatively correlated with the AES total score (r = −0.65, *p* = 0.0018, Fig. 1B). Additionally, the drift rate for Go trials (*v*._go_) was negatively correlated with AES total scores (r = −0.61 *p* = 0.0046, Fig. 1C) indicating that patients with a higher apathy score had a lower drift rate for Go trials, which reflects reduced processing efficiency and slower evidence accumulation (Table 2). No relations were observed between task-performance and the average *activity level*.

**Table 2:** Spearman correlations between apathy and motor activity measures and beta-weight contrast of task-related neural networks.

	r _(AES total)_	*p*	r _(Activity levels)_	*p*	r _(Activity variability)_	*p*
**Successful inhibition (No-Go correct > hit)**						
dAN	0.26	0.264	−0.17	0.523	−0.07	0.801
vAN	−0.58	**0.0072**	−0.01	0.955	−0.20	0.434
SMN	0.05	0.842	−0.04	0.866	−0.22	0.400
VN	0.07	0.781	−0.44	0.074	−0.36	0.162
FPN	−0.14	0.546	−0.40	0.112	−0.25	0.323
accSN	0.00	0.997	0.08	0.765	0.22	0.400
DMN1	−0.16	0.500	0.28	0.277	0.17	0.516
DMN2	−0.15	0.531	−0.01	0.978	0.13	0.613
DMN3	0.14	0.557	−0.17	0.510	−0.22	0.406
**Error inhibition (FA > hit)**						
dAN	0.26	0.266	−0.15	0.560	−0.36	0.158
vAN	0.00	1.000	−0.20	0.445	−0.50	**0.039**
SMN	0.38	0.096	−0.19	0.474	−0.12	0.633
VN	0.39	0.090	−0.15	0.554	−0.10	0.694
FPN	−0.01	0.972	−0.44	0.080	−0.29	0.252
accSN	−0.33	0.160	0.12	0.646	0.46	0.066
DMN1	−0.58	**0.0075**	0.17	0.504	0.02	0.926
DMN2	−0.07	0.778	−0.12	0.660	−0.09	0.736
DMN3	−0.08	0.737	−0.42	0.092	−0.43	0.088

*p*-values are derived from Spearman correlation analyses. Statistical significance was defined as *p* < 0.05. These correlations were presented without correction for multiple comparisons.

*dAN* dorsal attention network, *vAN* ventral attention network, *SMN* sensorimotor network, *VN* visual network, *FPN* frontoparietal network, *accSN* anterior cingulate cortex salience network, *DMN* default mode network, *AES total* the total scores of the apathy evaluation scale, *AC* activity counts, *Activity levels* the average value of motor activity measuring activity levels, *Activity variability* the root of mean squared successive difference of activity indicating activity variability.

### Task-relevant network connectivity

**Fig. 2:**
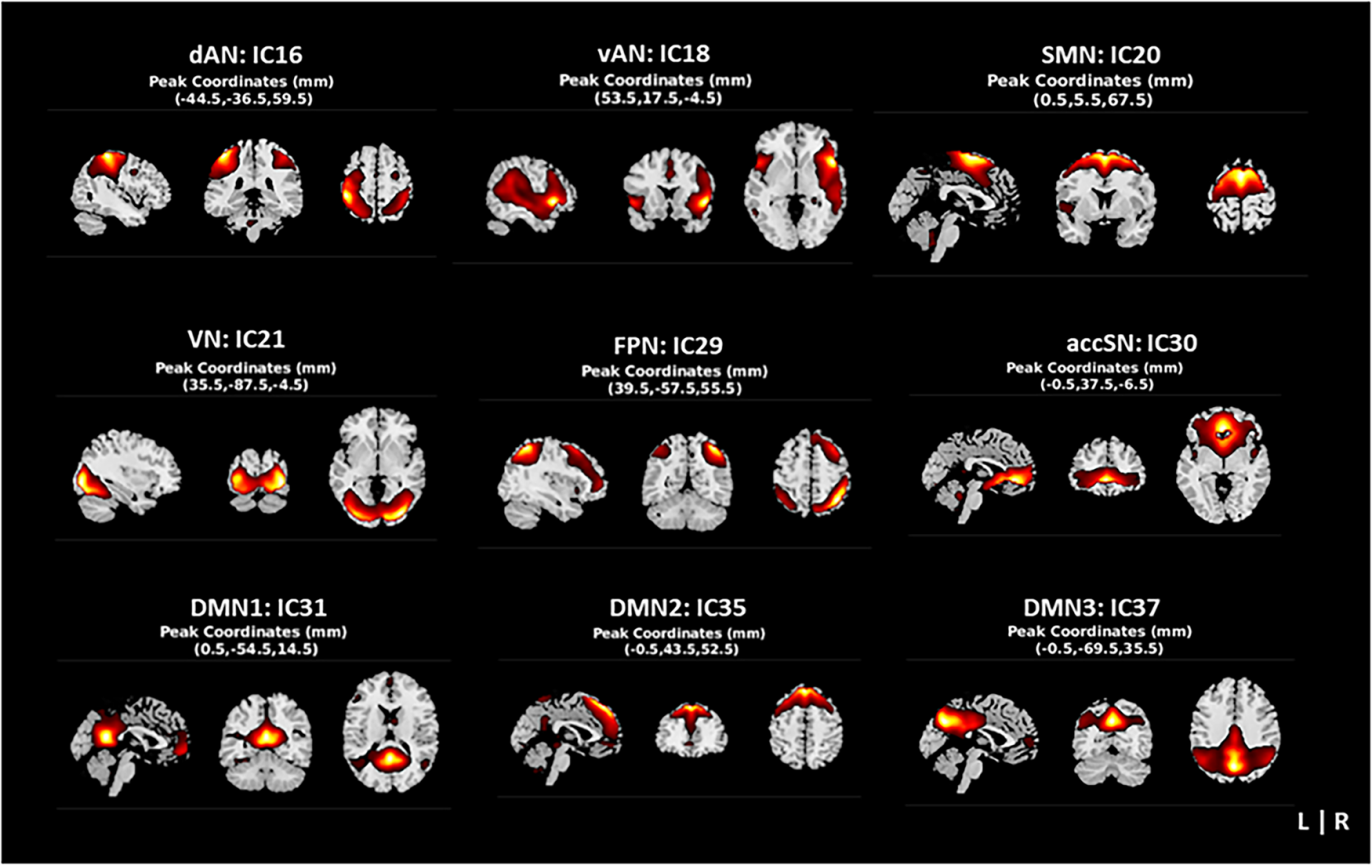
Task-relevant networks. Independent component 16 is identified as the dorsal attention network (dAN); Independent component 18 is identified as the ventral attention network (vAN); Independent component 20 is corresponding to the sensorimotor network (SMN); Independent component 21 is identified as the visual network (VN); Independent component 29 is identified as the frontoparietal network (FPN); Independent component 30 is corresponding to the anterior cingulate cortex salience network (accSN); Independent component 31, 35 and 37 are corresponding to the default mode network (DMN).

Forty-one independent components were generated after estimation using the MDL algorithm. The ICASSO approach was run twenty times to establish independent component stability, yielding an average intra-cluster stability index IQ above 0.75, suggesting good stability. After excluding 25 ICs reflecting artifacts such as head movement, respiratory motion, or heart pulsing, 16 functional networks were identified (see Fig. S2 in the supplement). The spatial sorting results of functional networks were provided in supplementary Table S2. Temporal sorting was conducted to detect the task-relevant components by modelling the three conditions (Hit, No-Go Correct, and False alarm), as well as the Go incorrect conditions and head movement parameters. One-sample t-tests on the beta-weights of each condition showed that nine networks significantly modulated by at least one task-specific condition, i.e., hit, No-Go correct or FA (see Table S3). Spatial maps of the task relevant networks were presented in Fig. 2.

### Associations between apathy, motor activity, performance, and network engagement during inhibition

**Fig. 3:**
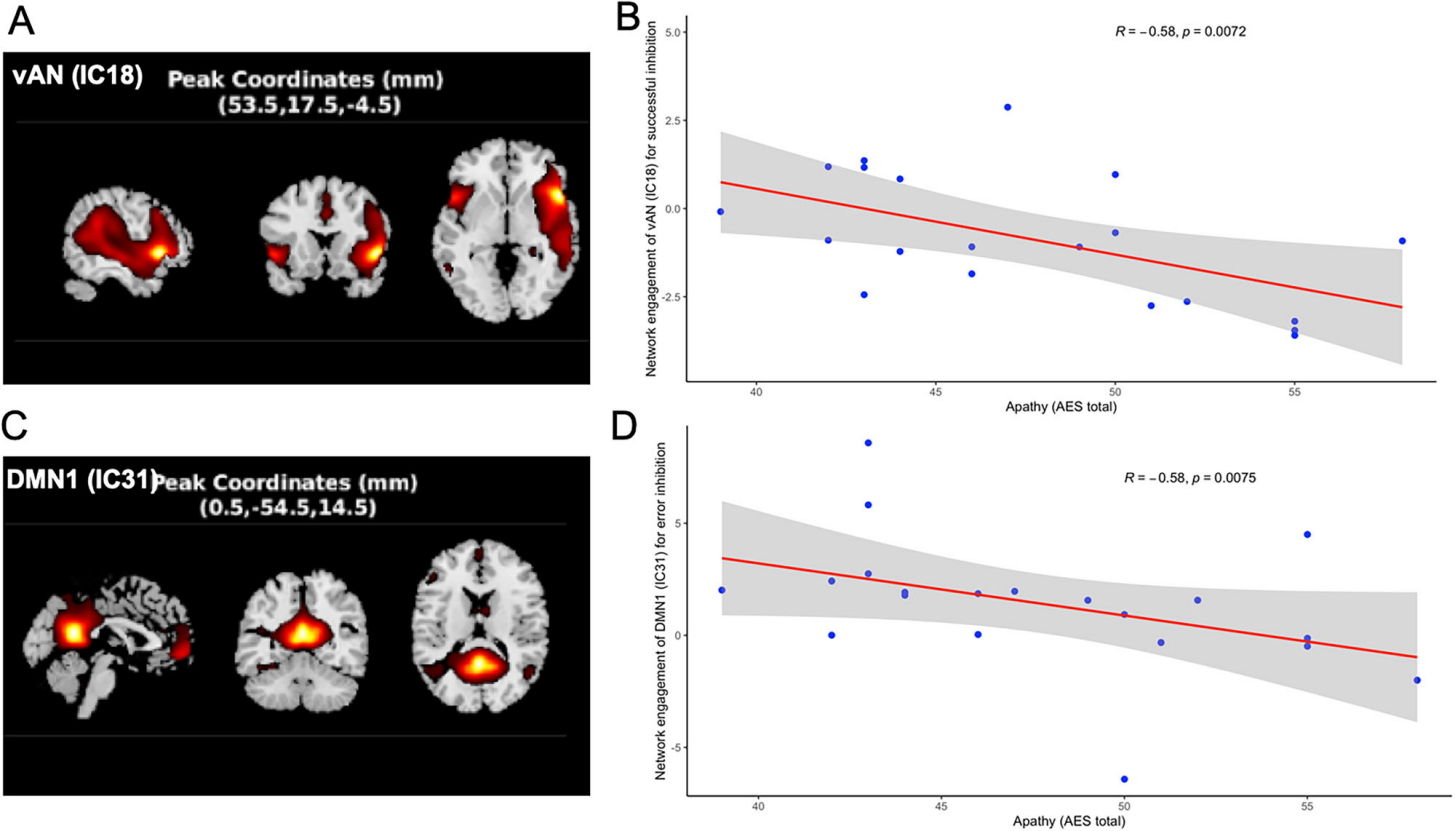
The relationship between apathy severity and network engagement measured with beta weights for task conditions. **A**, **B** Higher levels of apathy were related to a lower engagement of ventral attention network (vAN; i.e., independent component 18) for successful inhibition (i.e., correct No-Go > hit). **C**, **D** Higher levels of apathy were associated with a lower engagement of default mode network (DMN1; i.e., independent component 31) for error inhibition (i.e., false alarm > hit).

Spearman correlation analyses between apathy and the beta weights from task conditions showed that higher levels of apathy were related to a decreased engagement of ventral attention network (IC18) for successful inhibition (i.e., correct No-Go > hit; r = −0.58, *p* = 0.0072; Fig. 3A), a decreased engagement of DMN (IC31) for error inhibition (i.e., FA > hit; r = −0.58, *p* = 0.0075; Fig. 3B). Adding age and depression as covariates did not change the results for vAN (*p* < 0.05), however, the relation between apathy and DMN became non-significant (*p* = 0.12).

*Activity levels* were not related to network engagement on the task (Table 2). Lower motor *activity variability* was related to higher vAN engagement for error inhibition (r = −0.5, *p* = 0.039).

The overall accuracy of task was negatively correlated to the dorsal attention network (dAN) dAN (r = −0.57, *p* = 0.009), while positively correlated to accSN (r = 0.55, *p* = 0.011) for error inhibition (see Table S4). Model parameters (i.e., *ter, z* and *v*._nogo_) were related to network engagement of dAN for successful inhibition (*ps*: 0.015–0.029; see Table S5).

### Apathy associated within- and between- network connectivity irrespective of task-conditions

**Fig. 4:**
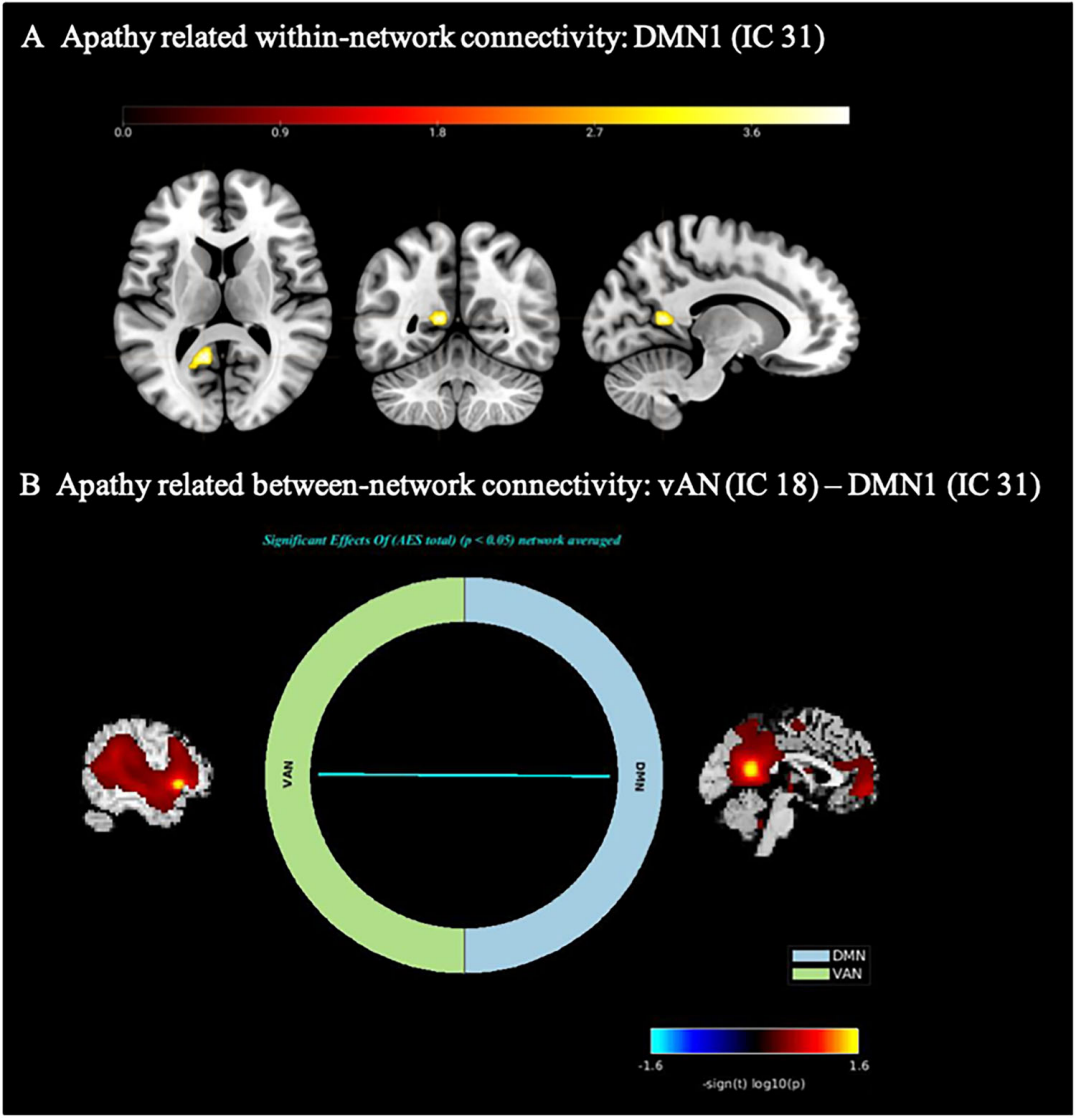
Apathy associated within- and between- network connectivity across the task conditions. **A** Higher severity of apathy was associated with increased within- network connectivity of a cluster at the left precuneus (cluster size k = 34, peak-coordinate [x = −12 y = −55 z = 11]) within default mode network (DMN1; corresponding to independent component 31). **B** More severe apathy was associated with lower between-network connectivity between ventral attention network (vAN; i.e., independent component 18) and default mode network (DMN1; i.e., independent component 31).

Regression analysis with apathy on the spatial maps revealed that AES total scores were positively associated with the left posterior cingulate cortex of the DMN1 network (IC 31; cluster size k = 34, peak-coordinate [x = −12 y = −55 z = 11], t = 4.22, *p*_FDR-corr_ = 0.034, Fig. 4A). In an exploratory analysis of connectivity between networks, apathy scores were negatively associated with functional network connectivity between the vAN and DMN1 networks (t = −2.47, *p* = 0.024, r² = 0.25; see Fig. 4B).

To examine whether actigraphy-measured activity levels and variability showed similar network associations as apathy, we conducted exploratory regressions testing relationships between motor activity and both within- and between-network connectivity during. Inhibitory control task. These analyses did not reveal any significant associations between motor activity and network connectivity.

## Discussion

This study investigated functional networks and cognitive processes underlying inhibitory control in relation to apathy among patients with schizophrenia exhibiting high levels of apathy. Higher apathy was associated with lower overall accuracy in response inhibition, more negative d′ values (indicating lower discrimination of stimuli and a response confusion), and low drift rates for the Go condition (indicating inefficiency in the evaluation of stimuli features and initiation of context-appropriate actions). Notably, diminished engagement of the vAN during successful inhibition, and reduced engagement of the DMN during error inhibition were linked to apathy in patients with schizophrenia. Stronger connectivity of the left posterior cingulate cortex within the DMN was associated with higher apathy symptom severity. Furthermore, reduced functional network connectivity between the vAN and DMN was associated with increased apathy in patients with schizophrenia. These findings suggest that inhibitory control performance and its neural underpinnings are relevant for understanding severity of apathy in the context of schizophrenia spectrum disorder.

The main result of our study was the finding that higher apathy related to decreased engagement of the vAN during successful inhibition [correct No-Go > hit] in patients with schizophrenia. Such inhibitory processing is a central aspect of cognitive control, which may be impaired in patients with schizophrenia and severe apathy. Consistent with this, we observed that the higher the patient’s level of apathy, the worse the performance in the response inhibition task. In healthy volunteers, the vAN, including the IFG, superior temporal gyrus, inferior and superior parietal lobule, has been involved in stopping-related behavior during an inhibition task [51]. IFG and temporoparietal junction (TPJ) recruitment has been shown in stimuli-driven attentional processing, such as detecting involuntary or unexpected stimuli, or responding to contextual cues [52]. Hypoactivation in the right IFG during response inhibition has been found in patients with schizophrenia compared with healthy controls [53, 54]. Furthermore, structural alterations of the prefrontal cortex have been reported to be associated with negative symptoms in patients with schizophrenia, including thinning of prefrontal cortex [55] and alterations of orbitofrontal cortex [56–58]. In the current study, patients with schizophrenia and high apathy exhibited lower vAN recruitment in No-Go trials than in Go trials. This may reflect deficits in adjusting neural activity resources according to inhibitory control demands [59]. However, behavioral model parameters (i.e., overall accuracy, *ter*, *z* and *v*._nogo_) were observed to correlate to the engagement of dAN (see Table S5), suggesting a potential interaction between the vAN and dAN in modulating behavioral performance [60].

On the behavioral level, higher apathy in patients with schizophrenia was linked to poor accuracy on the Go/No-Go task and a negative value of d’, indicating higher false alarm rate than hit rate and a lower ability to discriminate between stimuli. Decomposing the decision process, we observed that reduced drift rates for the Go condition were associated with higher apathy, reflecting inefficiency in evaluating stimuli features and selecting appropriate actions. This behavioural pattern is consistent with impulsivity, which has been shown to be associated with indecisiveness at the decision-making level [61]. Indecisiveness or decision inertia, characterized by exerting longer time deliberation and more effort for searching, may preclude individuals from deciding what goal-directed behavior to engage in, thereby, exacerbating apathy symptoms [62]. In addition, the high frequency of omission errors (i.e., missing responses to Go stimuli) suggests difficulties in initiating context-appropriate behaviours or developing prepotent responses, given that the frequency of Go trials is greater than the frequency of No-Go trials [53].

Reduced DMN engagement during failed inhibition (i.e., false alarm > hit) was also associated with higher apathy levels in patients with schizophrenia. The ACC and posterior cingulate cortex/precuneus, key nodes within the DMN, are critical for error processing in the Go/No-Go task [63], allowing individuals to monitor, evaluate, and adjust subsequent behavior to optimize overall performance [64]. Reduced involvement of DMN regions in patients with schizophrenia and higher apathy may affect error processing and behavioral optimization. It may hamper individuals to adapt their strategy in response to errors. A resting-state fMRI study in healthy individuals has shown functional connectivity of several regions within the DMN to be associated with self-efficacy [65]. Self-efficacy refers to a person’s beliefs in one’s ability to effectively perform the tasks needed to attain a valued goal. This is important for goal-directed behavior, and reduced recruitment in DMN in individuals with apathy likely contributes to impaired inhibitory task performance [66, 67]. However, the association between DMN recruitment and apathy became non-significant when accounting for depressive symptoms and age. This suggests that the observed DMN alterations may not be specific to apathy alone. Age-related changes in cognitive function and increased depressive symptoms could contribute to the relationship observed in the unadjusted analyses. Thus, DMN engagement may not reflect an independent neural correlate of apathy.

Our findings suggested that higher levels of apathy in patients with schizophrenia were associated with higher within-DMN network connectivity of the left posterior cingulate cortex and lower network connectivity between vAN and DMN across inhibitory control task conditions in patients with schizophrenia. This decoupling of attentional control regions in vAN from the DMN may underlie difficulties to reorient attention to dynamically changing stimuli in patients with schizophrenia and apathy, resulting in diminished behavioral response inhibition. Previous research has shown that DMN dysfunction was linked to a poor task performance in in patients with schizophrenia compared with healthy controls [23, 53]. We found weak connectivity between the vAN and DMN to be associated with higher apathy in patients with schizophrenia. This may suggest that the collaborative neural systems responsible for monitoring and reallocating cognitive resources in response to external stimuli are affected by patients with schizophrenia and higher apathy symptom severity.

Actigraphy-derived motor activity demonstrated a non-significant relationship with inhibitory control, though both were related to interview-measured apathy symptoms. This may indicate that motor activity and inhibitory control reflect distinct aspects of apathy. Spontaneous motor activity in daily life may be related to a loss of initiation of action and reward processing, while inhibitory control pertains more to cognitive domain of apathy [39, 68]. Actigraphy measured activity levels were presumed as an objective indicator of apathy underlying dissociable neural processes with interview-measured apathy during reward anticipation in schizophrenia [30]. Thus, motor activity may be closely related to motivational deficits in individuals with schizophrenia. Alternatively, the relatively brief duration of actigraphy assessment and the limited statistical power of the sample may underestimate the strength of the associations. Future research should investigate potential differential links between motor activity and the subdomains of apathy using larger samples and longer actigraphy assessment periods [69].

Several limitations of our study should be considered. First, we applied a broad functional network analysis approach rather than conventional neural response analysis on task contrast images of various brain regions. Thus, task activation in specific regions was not accounted for in our analysis. However, our approach allowed for insights at the network level, a biologically plausible way of investigating brain function, and further tests on ROI-based task activation were detailed in the supplement. Second, this study included a relatively small sample size of patients with schizophrenia and moderate to severe apathy symptoms, which limits statistical power and generalizability of the findings. However, there is still quite some variation in AES scores, with a range from 39 to 58, which supports the viability of our analyses. Smaller samples are more sensitive to individual variability and may inflate the risk of overfitting or identifying unstable connectivity patterns. In order to improving the robustness of multivariate analyses such as ICA and network connectivity, larger sample size (e.g., n ~ 30) and longer runs (e.g., >10 min) are advisable [70, 71]. Thus, although our multimodal results were convergent across behavioral, imaging, and actigraphy measures, the findings should be interpreted as preliminary. Replication in larger samples and including participants with lower levels of apathy is necessary). Third, the cross-sectional design limits our ability to infer causality regarding the associations on impaired inhibition with clinical apathy symptoms. Future studies could focus on the alterations in the vAN and DMN during inhibition to explore causal relationships with apathy. Additionally, the effects of medication, clinical characteristics, and disease progression on apathy in relation to inhibitory function should be considered in future research.

## Conclusion

The present study investigated the association between behaviour and neural network mechanisms of inhibitory control and apathy in patients with schizophrenia, providing novel insights into the relationships between apathy and connectivity of cognitive inhibitory control and attention networks during a Go/No-Go task. Apathy was related to poorer inhibitory control in patients with schizophrenia, shown by less recruitment of the vAN and DMN, and weak network connectivity between vAN and DMN. These findings suggest potential deficits in attention control and adjusting cognitive resources in response to external demands during inhibitory control in patients with schizophrenia and higher levels of apathy. Actigraphy measurements were related to apathy, but not to inhibitory control processing during the go/No-Go task. Interventions modulating the key regions in the vAN (including the temporoparietal junction and the ventrolateral prefrontal cortex) might help enhance inhibitory control and support cognitive flexibility for patients with schizophrenia with severe apathy.

## Supplementary information

Supplemental Material

## Data Availability

Data can be made available upon reasonable request from the corresponding author.
