# Supplementary material for: Inhibitory control deficits and ventral attention network alterations linked to apathy in schizophrenia
Source: Transl Psychiatry. 2026 May 21;16:361. doi: 10.1038/s41398-026-04066-7 (PMC13369803; doi:10.1038/s41398-026-04066-7)
Supplement: Supplementary file 1 — Supplemental Material [file 41398_2026_4066_MOESM1_ESM.docx]

Supplementary Material

[Supplementary Text 2](#_Toc226061111)

[Text S1 MRI acquisition parameters 2](#_Toc226061112)

[Text S2 ROI-based activation analyses 2](#_Toc226061113)

[Supplementary Tables 3](#_Toc226061114)

[Table S1 DDM Parameters and cognitive implications 3](#_Toc226061115)

[Table S2 Spatial sorting results with multiple regressions for the independent component on the network template 3](#_Toc226061116)

[Table S3 Temporal sorting results with one sample t tests on beta weights of task-relevant networks 4](#_Toc226061117)

[Table S4 Spearman correlations between Go/No-Go performance and beta-weight contrast of task-related neural networks. 5](#_Toc226061118)

[Table S5 Spearman correlations between model parameters and beta-weight contrast of task-related neural networks. 6](#_Toc226061119)

[Table S6 Spearman correlations between apathy measures and Region of Interest activations (ROIs) 7](#_Toc226061120)

[Table S7 Spearman correlations between model parameters and Region of Interest activations (ROIs). 8](#_Toc226061121)

[Table S8 Spearman correlations between Go/No-Go performance and Region of Interest activations (ROIs). 9](#_Toc226061122)

[Supplementary Figures 13](#_Toc226061123)

[Figure S1 Distribution of levels of apathy as measured by AES total score. The participants were selected based on a cutoff score of AES-apathy subscale of 27 indicating clinical apathy. 13](#_Toc226061124)

[Figure S2 16 Networks identified through visual selection on the spatial map and spectral power, after excluding 24 ICs reflects artifacts. 13](#_Toc226061125)

##

## **Supplementary Text**

### Text S1 MRI acquisition parameters

The MRI data were acquired using a 3.0 Tesla Philips MRI scanner (Philips Medical Systems, Best, NL), equipped with a 32-channel SENSE head coil. A T2*-weighted echo planar imaging sequence (47 descending axial slices; slice thickness = 3mm; TR = 2000ms; TE = 22ms; FOV = 192*141*192mm; voxel size = 3*3*3mm, 360 volumes) was used to obtain functional images during the Go/No-Go task. High-resolution structural images were acquired using fast-field echo (170 axial slices; TR = 9ms; TE = 3.55ms; FOV: 232*256; 256*256; voxel size = 1*1*1mm).

### Text S2 ROI-based activation analyses

To complement our network-level analyses, we performed an exploratory GLM-based activation analysis of Go/No-Go task contrasts (i.e., successful inhibition: No-Go correct > Hit; error inhibition: False alarm > Hit). We identified a set of regions of interest coordinates for each contrast based on previous literature (Criaud and Boulinguez, 2013; Weigard et al., 2020). Eleven ROIs for the successful inhibition contrast were defined and eight ROIs were used for the error inhibition contrast with a 8-mm-radius spheres by using MarsBar toolbox (Brett et al., 2010). The ROIs for the successful inhibition included the left inferior front-35 (-35, 21, -9),left middle frontal (-46, 25, 25), left supramarginal (-59, -50, 35), medial frontal (2, 22, 41), left medial frontal (-38, 59, 3), right inferior frontal (51, 14, 25), right inferior frontal (39, 24, -10), right inferior parietal (51, -50, 41), right middle frontal (46, 39, 24), right superior frontal (13, 18, 58), and right superior temporal (63, -20, -5) regions. The ROIs for the error inhibition included the anterior cingulate (0, 22, 38), left insula (-38, 20, -6), left parietal (-62, -44, 34), left striatum (-12, 10, -10), right insula (42, 18, -6), right parietal (58, -44, 30), right striatum (14, 10, -10), right pre-SMA (4, 30, 54) regions. The activations within ROIs for each contrast of each participant were extracted. Spearman correlation analyses were performed to assess the associations between the ROI-based neural activation with apathy scores, motor activity and behavioral and computational parameters of the Go/No-Go inhibition task using R software. Results were considered significant at *p*<.05, two-tailed, and uncorrected.

## **Supplementary Tables**

### Table S1 DDM Parameters and cognitive implications

| **Parameters** | **Profile** | **Potential implications** | **influenced by** |
| --- | --- | --- | --- |
| Drift rate (v) | The speed with which the accumulation process approaches one of the two boundaries | Represent the relative evidence for or against a particular response;  Purer measure of processing efficiency | Task difficulty/ familiarity;  Noise;  DA medication |
| Decision threshold (a) | The distance between the two boundaries | How much evidence must be accumulated until a response is executed; the caution of response | Context (accuracy or speed) |
| Non-decision time (ter) | The time of perception, movement initiation, and execution | Time for stimulus encoding and response execution | Reactivity; arousal levels; attention allocation |
| Starting-point bias (z) | The starting point of the drift process relative to the two boundaries | Response bias | Impulsivity |

### Table S2 Spatial sorting results with multiple regressions for the independent component on the network template

| **IC** | **Network** | **Regression coefficient** |
| --- | --- | --- |
| 16 | dAN | 0.18 |
| 18 | vAN | 0.14 |
| 20 | SN | 0.13 |
| 21 | Visual network | 0.16 |
| 29 | FPN | 0.17 |
| 30 | accSN | 0.09 |
| 31 | DMN | 0.19 |
| 35 | dDMN | 0.12 |
| 37 | pDMN | 0.27 |

### Table S3 Temporal sorting results with one sample t tests on beta weights of task-relevant networks

| **Network** | **IC** | **Hit** | | **No-Go correct** | | **False alarm** | |
| --- | --- | --- | --- | --- | --- | --- | --- |
|  |  | t | *p* | t | *p* | t | *p* |
| dAN | 16 | -4.67 | 0.0002 *** | -3.52 | 0.002 ** | -6.34 | 4.42e-06 *** |
| vAN | 18 | -2.65 | 0.016 * | -3.75 | 0.001 ** | -1.68 | 0.109 |
| SMN | 20 | -5.56 | 2.32e-05 *** | -2.87 | 0.01 * | -4.36 | 0.0003 *** |
| VN | 21 | -6.88 | 1.46e-06 *** | -4.37 | 0.0003 *** | -5.83 | 1.28e-05 *** |
| FPN | 29 | -3.37 | 0.003 ** | 4.21 | 0.0005 *** | -2.09 | 0.05 |
| accSN | 30 | 2.04 | 0.056 | 2.19 | 0.042 * | 1.87 | 0.08 |
| DMN1 | 31 | 3.55 | 0.002 ** | 4.76 | 0.0001 *** | 2.31 | 0.03 * |
| DMN2 | 35 | 1.60 | 0.126 | 3.41 | 0.003 ** | 3.30 | 0.004 ** |
| DMN3 | 37 | 3.30 | 0.004 ** | 2.47 | 0.02 * | 2.56 | 0.019 * |

Note: IC=independent component; dAN=dorsal attention network; vAN=ventral attention network; SMN= sensorimotor network; VN=visual network; FPN=frontoparietal network; accSN= anterior cingulate cortex salience network; DMN=default mode network

### Table S4 Spearman correlations between Go/No-Go performance and beta-weight contrast of task-related neural networks.

|  | ***r (overall accuracy)*** | ***p*** | ***r (hit_rate)*** | ***p*** | ***r (FA_rate)*** | ***p*** | ***r (hit_RT)*** | ***p*** | ***r (FA_RT)*** | ***p*** | ***r (d’)*** | ***p*** |
| --- | --- | --- | --- | --- | --- | --- | --- | --- | --- | --- | --- | --- |
| **Successful inhibition (No-Go correct > hit)** | | | |  |  |  |  |  |  |  |  |  |
| dAN | -0.09 | .702 | 0.17 | .479 | 0.63 | **.003 **** | -0.45 | **.045 *** | -0.44 | .054 | -0.34 | .148 |
| vAN | 0.12 | .615 | 0.03 | .897 | -0.25 | .282 | 0.11 | .636 | 0.24 | .301 | 0.18 | .435 |
| SMN | -0.09 | .695 | 0.11 | .631 | 0.30 | .193 | -0.27 | .243 | -0.19 | .427 | -0.24 | .301 |
| VN | -0.09 | .695 | 0.02 | .950 | 0.23 | .319 | -0.06 | .816 | -0.07 | .758 | -0.13 | .574 |
| FPN | 0.15 | .526 | 0.15 | .537 | 0.01 | .957 | -0.12 | .600 | 0.04 | .860 | 0.15 | .519 |
| accSN | 0.17 | .473 | 0.16 | .504 | -0.22 | .360 | 0.11 | .650 | 0.15 | .523 | 0.21 | .376 |
| DMN1 | -0.14 | .562 | -0.19 | .435 | -0.14 | .552 | 0.14 | .552 | 0.07 | .767 | 0.09 | .719 |
| DMN2 | 0.02 | .927 | -0.07 | .755 | -0.12 | .627 | 0.24 | .310 | -0.01 | .970 | 0.17 | .470 |
| DMN3 | -0.41 | .073 | -0.29 | .223 | -0.10 | .689 | 0.26 | .271 | 0.17 | .482 | -0.26 | .268 |
| **Error inhibition (FA > hit)** | | | |  |  |  |  |  |  |  |  |  |
| dAN | -0.57 | **.009 **** | -0.60 | **.005**** | -0.14 | .565 | 0.43 | .056 | 0.41 | .076 | -0.33 | .158 |
| vAN | -0.22 | .350 | -0.11 | .647 | 0.09 | .710 | -0.01 | .970 | 0.06 | .801 | -0.32 | .169 |
| SMN | -0.26 | .273 | -0.22 | .352 | 0.13 | .595 | 0.04 | .855 | 0.10 | .677 | -0.15 | .523 |
| VN | -0.15 | .522 | -0.10 | .684 | 0.13 | .580 | 0.08 | .724 | 0.01 | .980 | -0.09 | .710 |
| FPN | 0.06 | .813 | 0.09 | .707 | 0.29 | .219 | -0.20 | .387 | -0.13 | .587 | -0.07 | .772 |
| accSN | 0.55 | **.011 *** | 0.42 | .069 | 0.04 | .862 | -0.33 | .150 | -0.23 | .323 | 0.47 | **.036 *** |
| DMN1 | 0.30 | .197 | 0.18 | .438 | -0.19 | .421 | -0.09 | .715 | 0.09 | .715 | 0.36 | .121 |
| DMN2 | 0.20 | .397 | 0.28 | .226 | 0.08 | .748 | -0.19 | .427 | -0.11 | .654 | -0.04 | .875 |
| DMN3 | 0.15 | .535 | 0.24 | .299 | 0.13 | .593 | -0.20 | .394 | -0.06 | .796 | -0.06 | .816 |

### Table S5 Spearman correlations between model parameters and beta-weight contrast of task-related neural networks.

|  | ***r (v.go)*** | ***p*** | ***r (a)*** | ***p*** | ***r (ter)*** | ***p*** | ***r (z)*** | ***p*** | ***r (v.nogo)*** | ***p*** |
| --- | --- | --- | --- | --- | --- | --- | --- | --- | --- | --- |
| **Successful inhibition (No-Go correct > hit)** | | | | | |  |  |  |  |  |
| dAN | -0.31 | .182 | 0.44 | .051 | -0.54 | **.015 *** | 0.50 | **.024 *** | 0.49 | **.029 *** |
| vAN | 0.22 | .342 | 0.00 | .990 | 0.16 | .506 | -0.14 | .556 | -0.19 | .431 |
| SMN | -0.13 | .591 | 0.14 | .552 | -0.23 | .332 | 0.19 | .424 | 0.09 | .710 |
| VN | -0.23 | .329 | 0.23 | .323 | -0.11 | .654 | 0.25 | .295 | 0.29 | .217 |
| FPN | 0.16 | .494 | 0.40 | .077 | -0.22 | .346 | 0.16 | .502 | 0.07 | .772 |
| accSN | 0.22 | .346 | -0.08 | .753 | 0.11 | .654 | -0.09 | .719 | -0.35 | .130 |
| DMN1 | -0.08 | .724 | -0.26 | .268 | 0.16 | .502 | -0.31 | .191 | 0.09 | .691 |
| DMN2 | 0.07 | .767 | -0.22 | .352 | 0.26 | .259 | -0.18 | .446 | 0.01 | .975 |
| DMN3 | -0.23 | .336 | -0.37 | .113 | 0.26 | .277 | -0.29 | .217 | 0.08 | .748 |
| **Error inhibition (FA > hit)** | | | | |  |  |  |  |  |  |
| dAN | -0.44 | .054 | -0.19 | .420 | 0.26 | .277 | -0.32 | .171 | 0.31 | .184 |
| vAN | -0.23 | .329 | -0.02 | .950 | -0.08 | .729 | -0.04 | .875 | 0.22 | .352 |
| SMN | -0.14 | .565 | 0.12 | .622 | -0.11 | .654 | -0.02 | .950 | 0.29 | .217 |
| VN | -0.03 | .910 | -0.04 | .875 | 0.03 | .915 | 0.04 | .860 | 0.02 | .940 |
| FPN | -0.02 | .950 | 0.25 | .292 | -0.26 | .274 | 0.21 | .380 | 0.36 | .120 |
| accSN | 0.51 | **.020 *** | 0.27 | .248 | -0.22 | .346 | 0.27 | .246 | -0.28 | .232 |
| DMN1 | 0.31 | .184 | 0.09 | .710 | -0.10 | .677 | -0.08 | .743 | -0.31 | .186 |
| DMN2 | 0.04 | .880 | 0.28 | .227 | -0.18 | .439 | 0.27 | .246 | -0.06 | .796 |
| DMN3 | 0.00 | .990 | 0.31 | .179 | -0.24 | .310 | 0.29 | .220 | 0.02 | .925 |

### Table S6 Spearman correlations between apathy measures and Region of Interest activations (ROIs)

|  | **AES-total** | |  |  | **Activity levels** | |  |  | **Activity variability** | |  |  |
| --- | --- | --- | --- | --- | --- | --- | --- | --- | --- | --- | --- | --- |
|  | *r* | [95% | CI] | *p* | *r* | [95% | CI] | *p* | *r* | [95% | CI] | *p* |
| **Successful inhibition (No-Go correct > hit)** |  |  |  |  |  |  |  |  |  |  |  |  |
| 1SI L. Inferior Frontal (-35, 21, -9) | -0.36 | [-0.69 | 0.10] | .118 | 0.10 | [-0.40 | 0.56] | .694 | 0.07 | [-0.42 | 0.53] | .786 |
| 2SI L. Middle Frontal (-46, 25, 25) | 0.10 | [-0.36 | 0.52] | .685 | -0.09 | [-0.54 | 0.41] | .743 | -0.23 | [-0.64 | 0.28] | .368 |
| 3SI L. Supramarginal (-59, -50, 35) | -0.28 | [-0.65 | 0.18] | .224 | -0.15 | [-0.59 | 0.36] | .573 | -0.32 | [-0.70 | 0.19] | .205 |
| 4SI Medial Frontal (2, 22, 41) | 0.23 | [-0.23 | 0.61] | .322 | -0.09 | [-0.55 | 0.41] | .722 | -0.20 | [-0.62 | 0.31] | .451 |
| 5SI L. Medial Frontal (-38, 59, 3) | 0.00 | [-0.44 | 0.45] | .985 | -0.22 | [-0.63 | 0.29] | .395 | -0.22 | [-0.63 | 0.29] | .400 |
| 6SI R. Inferior Frontal (51, 14, 25) | -0.11 | [-0.53 | 0.35] | .637 | -0.43 | [-0.75 | 0.07] | .086 | -0.46 | [-0.77 | 0.03] | .064 |
| 7SI R. Inferior Frontal (39, 24, -10) | 0.14 | [-0.32 | 0.55] | .561 | 0.03 | [-0.46 | 0.50] | .918 | 0.04 | [-0.45 | 0.51] | .874 |
| 8SI R. Inferior Parietal (51, -50, 41) | 0.10 | [-0.36 | 0.52] | .673 | -0.24 | [-0.64 | 0.27] | .358 | -0.10 | [-0.55 | 0.40] | .708 |
| 9SI R. Middle Frontal (46, 39, 24) | 0.23 | [-0.23 | 0.61] | .319 | -0.21 | [-0.63 | 0.30] | .417 | -0.18 | [-0.61 | 0.33] | .498 |
| 10SI R. Superior Frontal (13, 18, 58) | 0.06 | [-0.40 | 0.49] | .815 | 0.12 | [-0.38 | 0.57] | .639 | 0.08 | [-0.41 | 0.54] | .751 |
| 11SI R. Superior Temporal (63, -20, -5) | 0.04 | [-0.41 | 0.48] | .862 | -0.09 | [-0.55 | 0.41] | .736 | -0.15 | [-0.59 | 0.36] | .573 |
| **Error inhibition (FA > hit)** |  |  |  |  |  |  |  |  |  |  |  |  |
| 12EI Anterior Cingulate (0, 22, 38) | 0.41 | [-0.04 | 0.72] | .070 | -0.31 | [-0.69 | 0.20] | .224 | -0.43 | [-0.76 | 0.06] | .082 |
| 13EI L. Insula/IFG (-38, 20, -6) | -0.10 | [-0.52 | 0.36] | .685 | 0.14 | [-0.36 | 0.58] | .580 | -0.03 | [-0.51 | 0.45] | .896 |
| 14EI L. Parietal (-62, -44, 34) | 0.06 | [-0.39 | 0.49] | .786 | -0.24 | [-0.65 | 0.27] | .353 | -0.37 | [-0.72 | 0.14] | .149 |
| 15EI L. Striatum (-12, 10, -10) | -0.27 | [-0.63 | 0.20] | .259 | 0.03 | [-0.46 | 0.50] | .911 | -0.06 | [-0.53 | 0.43] | .815 |
| 16EI R. Insula/IFG (42, 18, -6) | 0.18 | [-0.29 | 0.57] | .456 | -0.02 | [-0.50 | 0.47] | .940 | -0.10 | [-0.55 | 0.40] | .708 |
| 17EI R. Parietal (58, -44, 30) | 0.08 | [-0.38 | 0.51] | .735 | -0.07 | [-0.53 | 0.42] | .786 | -0.31 | [-0.69 | 0.20] | .228 |
| 18EI R. Striatum (14, 10, -10) | 0.15 | [-0.31 | 0.56] | .529 | 0.01 | [-0.47 | 0.49] | .970 | -0.16 | [-0.59 | 0.35] | .548 |
| 19EI R. pre-SMA (4, 30, 54) | 0.10 | [-0.36 | 0.52] | .669 | -0.30 | [-0.68 | 0.21] | .236 | -0.51 | [-0.80 | -0.04] | **.037** |

### Table S7 Spearman correlations between model parameters and Region of Interest activations (ROIs).

|  | ***v.go*** |  |  |  | ***a*** |  |  |  | ***ter*** |  |  |  | ***z*** |  |  |  | ***v.nogo*** |  |  |  |
| --- | --- | --- | --- | --- | --- | --- | --- | --- | --- | --- | --- | --- | --- | --- | --- | --- | --- | --- | --- | --- |
|  | ***r*** | [95% | CI] | *p* | ***r*** | [95% | CI] | *p* | *r* | [95% | CI] | *p* | ***r*** | [95% | CI] | *p* | *r* | [95% | CI] | *p* |
| **Successful inhibition (No-Go correct > hit)** |  |  |  |  |  |  |  |  |  |  |  |  |  |  |  |  |  |  |  |  |
| 1SI L. Inferior Frontal (-35, 21, -9) | 0.12 | [-0.34 | 0.53] | .627 | -0.01 | [-0.45 | 0.43] | .955 | -0.04 | [-0.47 | 0.41] | .880 | 0.13 | [-0.33 | 0.54] | .582 | -0.07 | [-0.49 | 0.39] | .782 |
| 2SI L. Middle Frontal (-46, 25, 25) | -0.32 | [-0.67 | 0.14] | .171 | 0.05 | [-0.40 | 0.48] | .830 | -0.18 | [-0.57 | 0.29] | .458 | 0.07 | [-0.38 | 0.50] | .758 | 0.30 | [-0.17 | 0.65] | .205 |
| 3SI L. Supramarginal (-59, -50, 35) | -0.07 | [-0.50 | 0.38] | .762 | -0.20 | [-0.59 | 0.27] | .398 | 0.18 | [-0.29 | 0.57] | .454 | -0.14 | [-0.55 | 0.32] | .544 | -0.03 | [-0.47 | 0.41] | .885 |
| 4SI Medial Frontal (2, 22, 41) | -0.43 | [-0.73 | 0.02] | .059 | 0.10 | [-0.36 | 0.52] | .673 | -0.24 | [-0.62 | 0.23] | .307 | 0.30 | [-0.17 | 0.65] | .205 | 0.35 | [-0.11 | 0.68] | .135 |
| 5SI L. Medial Frontal (-38, 59, 3) | -0.27 | [-0.64 | 0.19] | .246 | -0.08 | [-0.50 | 0.38] | .753 | 0.10 | [-0.36 | 0.52] | .682 | -0.12 | [-0.53 | 0.34] | .622 | 0.21 | [-0.26 | 0.60] | .376 |
| 6SI R. Inferior Frontal (51, 14, 25) | -0.19 | [-0.58 | 0.27] | .420 | 0.10 | [-0.36 | 0.52] | .682 | -0.07 | [-0.50 | 0.38] | .762 | -0.00 | [-0.45 | 0.44] | .985 | 0.31 | [-0.15 | 0.66] | .179 |
| 7SI R. Inferior Frontal (39, 24, -10) | -0.29 | [-0.65 | 0.18] | .222 | 0.27 | [-0.20 | 0.64] | .248 | -0.40 | [-0.71 | 0.06] | .083 | 0.31 | [-0.15 | 0.66] | .182 | 0.32 | [-0.14 | 0.67] | .164 |
| 8SI R. Inferior Parietal (51, -50, 41) | -0.21 | [-0.60 | 0.26] | .373 | 0.06 | [-0.39 | 0.49] | .801 | -0.04 | [-0.47 | 0.41] | .875 | -0.07 | [-0.50 | 0.39] | .777 | 0.26 | [-0.21 | 0.63] | .274 |
| 9SI R. Middle Frontal (46, 39, 24) | -0.39 | [-0.71 | 0.07] | .092 | 0.35 | [-0.11 | 0.69] | .128 | -0.44 | [-0.74 | 0.00] | .051 | 0.40 | [-0.05 | 0.72] | .079 | 0.54 | [0.12 | 0.79] | **.015** |
| 10SI R. Superior Frontal (13, 18, 58) | -0.08 | [-0.51 | 0.37] | .724 | 0.13 | [-0.33 | 0.54] | .578 | -0.33 | [-0.67 | 0.14] | .160 | 0.12 | [-0.34 | 0.54] | .609 | 0.02 | [-0.42 | 0.46] | .920 |
| 11SI R. Superior Temporal (63, -20, -5) | -0.21 | [-0.60 | 0.25] | .366 | 0.12 | [-0.34 | 0.54] | .600 | -0.27 | [-0.64 | 0.19] | .246 | 0.13 | [-0.33 | 0.54] | .596 | 0.42 | [-0.02 | 0.73] | .062 |
| **Error inhibition (FA > hit)** |  |  |  |  |  |  |  |  |  |  |  |  |  |  |  |  |  |  |  |  |
| 12EI Anterior Cingulate (0, 22, 38) | -0.51 | [-0.78 | -0.09] | **.022** | -0.02 | [-0.46 | 0.43] | .945 | -0.01 | [-0.45 | 0.43] | .965 | 0.04 | [-0.41 | 0.47] | .880 | 0.42 | [-0.03 | 0.72] | .069 |
| 13EI L. Insula/IFG (-38, 20, -6) | -0.05 | [-0.48 | 0.41] | .850 | 0.24 | [-0.23 | 0.62] | .310 | -0.28 | [-0.65 | 0.18] | .225 | 0.21 | [-0.25 | 0.60] | .366 | 0.18 | [-0.28 | 0.58] | .435 |
| 14EI L. Parietal (-62, -44, 34) | -0.28 | [-0.64 | 0.18] | .230 | 0.12 | [-0.34 | 0.54] | .600 | -0.07 | [-0.49 | 0.39] | .782 | -0.00 | [-0.44 | 0.44] | .990 | 0.34 | [-0.12 | 0.68] | .141 |
| 15EI L. Striatum (-12, 10, -10) | -0.27 | [-0.63 | 0.20] | .257 | -0.01 | [-0.45 | 0.43] | .955 | 0.08 | [-0.38 | 0.50] | .743 | 0.08 | [-0.38 | 0.50] | .753 | 0.24 | [-0.23 | 0.61] | .316 |
| 16EI R. Insula/IFG (42, 18, -6) | -0.25 | [-0.62 | 0.22] | .298 | 0.18 | [-0.28 | 0.58] | .446 | -0.23 | [-0.61 | 0.24] | .339 | 0.06 | [-0.39 | 0.49] | .806 | 0.50 | [0.08 | 0.77] | **.024** |
| 17EI R. Parietal (58, -44, 30) | -0.33 | [-0.67 | 0.13] | .158 | 0.05 | [-0.41 | 0.48] | .850 | -0.19 | [-0.58 | 0.28] | .431 | -0.01 | [-0.45 | 0.43] | .955 | 0.31 | [-0.15 | 0.66] | .179 |
| 18EI R. Striatum (14, 10, -10) | -0.57 | [-0.81 | -0.18] | **.008** | -0.28 | [-0.65 | 0.18] | .225 | 0.27 | [-0.19 | 0.64] | .246 | -0.12 | [-0.54 | 0.34] | .600 | 0.28 | [-0.19 | 0.64] | .240 |
| 19EI R. pre-SMA (4, 30, 54) | -0.29 | [-0.65 | 0.18] | .220 | -0.09 | [-0.51 | 0.37] | .705 | 0.07 | [-0.39 | 0.50] | .777 | -0.15 | [-0.55 | 0.32] | .539 | 0.31 | [-0.16 | 0.66] | .188 |

### Table S8 Spearman correlations between Go/No-Go performance and Region of Interest activations (ROIs).

|  | ***overall ACC*** |  |  |  | ***Hit rate*** |  |  |  | ***FA rate*** |  |  |  | ***Hit RT*** |  |  |  | ***FA RT*** |  |  |  | ***d’*** |  |  |  |
| --- | --- | --- | --- | --- | --- | --- | --- | --- | --- | --- | --- | --- | --- | --- | --- | --- | --- | --- | --- | --- | --- | --- | --- | --- |
|  | ***r*** | [95% | CI] | *p* | ***r*** | [95% | CI] | *p* | *r* | [95% | CI] | *p* | ***r*** | [95% | CI] | *p* | *r* | [95% | CI] | *p* | *r* | [95% | CI] | *p* |
| **Successful inhibition (No-Go correct > hit)** |  |  |  |  |  |  |  |  |  |  |  |  |  |  |  |  |  |  |  |  |  |  |  |  |
| 1SI L. Inferior Frontal (-35, 21, -9) | 0.17 | [-0.30 | 0.57] | .485 | 0.24 | [-0.23 | 0.62] | .310 | 0.18 | [-0.29 | 0.57] | .454 | -0.21 | [-0.60 | 0.25] | .369 | -0.26 | [-0.63 | 0.21] | .268 | 0.09 | [-0.37 | 0.51] | .705 |
| 2SI L. Middle Frontal (-46, 25, 25) | -0.36 | [-0.69 | 0.09] | .114 | -0.23 | [-0.61 | 0.23] | .319 | 0.22 | [-0.24 | 0.61] | .347 | -0.15 | [-0.56 | 0.31] | .514 | -0.16 | [-0.57 | 0.30] | .490 | -0.38 | [-0.71 | 0.07] | .094 |
| 3SI L. Supramarginal (-59, -50, 35) | -0.11 | [-0.52 | 0.35] | .658 | -0.13 | [-0.54 | 0.33] | .584 | -0.04 | [-0.48 | 0.41] | .862 | 0.13 | [-0.33 | 0.54] | .578 | 0.06 | [-0.39 | 0.49] | .796 | 0.02 | [-0.42 | 0.46] | .920 |
| 4SI Medial Frontal (2, 22, 41) | -0.35 | [-0.68 | 0.11] | .136 | -0.06 | [-0.49 | 0.39] | .794 | 0.49 | [0.07 | 0.77] | **.027** | -0.28 | [-0.64 | 0.19] | .232 | -0.37 | [-0.70 | 0.09] | .112 | -0.56 | [-0.80 | -0.15] | **.011** |
| 5SI L. Medial Frontal (-38, 59, 3) | -0.29 | [-0.65 | 0.18] | .218 | -0.27 | [-0.64 | 0.19] | .241 | -0.06 | [-0.49 | 0.39] | .791 | 0.13 | [-0.33 | 0.54] | .578 | 0.13 | [-0.33 | 0.54] | .582 | -0.16 | [-0.56 | 0.31] | .510 |
| 6SI R. Inferior Frontal (51, 14, 25) | -0.12 | [-0.53 | 0.34] | .617 | -0.10 | [-0.52 | 0.36] | .670 | 0.02 | [-0.43 | 0.46] | .942 | 0.00 | [-0.44 | 0.44] | .995 | 0.16 | [-0.30 | 0.56] | .498 | -0.13 | [-0.54 | 0.33] | .587 |
| 7SI R. Inferior Frontal (39, 24, -10) | -0.23 | [-0.61 | 0.23] | .319 | 0.05 | [-0.40 | 0.48] | .830 | 0.45 | [0.01 | 0.74] | **.046** | -0.41 | [-0.72 | 0.04] | .073 | -0.36 | [-0.69 | 0.09] | .115 | -0.44 | [-0.74 | -0.00] | .050 |
| 8SI R. Inferior Parietal (51, -50, 41) | -0.27 | [-0.64 | 0.20] | .253 | -0.18 | [-0.57 | 0.29] | .456 | 0.01 | [-0.44 | 0.45] | .970 | 0.08 | [-0.38 | 0.50] | .753 | 0.12 | [-0.34 | 0.53] | .622 | -0.21 | [-0.60 | 0.25] | .369 |
| 9SI R. Middle Frontal (46, 39, 24) | -0.20 | [-0.59 | 0.26] | .388 | 0.04 | [-0.41 | 0.48] | .862 | 0.53 | [0.11 | 0.79] | **.016** | -0.39 | [-0.71 | 0.06] | .086 | -0.37 | [-0.70 | 0.09] | .112 | -0.36 | [-0.69 | 0.09] | .115 |
| 10SI R. Superior Frontal (13, 18, 58) | -0.13 | [-0.54 | 0.33] | .580 | -0.01 | [-0.45 | 0.44] | .970 | 0.19 | [-0.27 | 0.58] | .418 | -0.34 | [-0.68 | 0.12] | .137 | -0.22 | [-0.60 | 0.25] | .352 | -0.20 | [-0.59 | 0.27] | .409 |
| 11SI R. Superior Temporal (63, -20, -5) | -0.26 | [-0.63 | 0.21] | .273 | -0.10 | [-0.52 | 0.35] | .661 | 0.27 | [-0.20 | 0.64] | .251 | -0.25 | [-0.62 | 0.22] | .295 | -0.23 | [-0.61 | 0.24] | .332 | -0.39 | [-0.71 | 0.07] | .092 |
| **Error inhibition (FA > hit)** |  |  |  |  |  |  |  |  |  |  |  |  |  |  |  |  |  |  |  |  |  |  |  |  |
| 12EI Anterior Cingulate (0, 22, 38) | -0.50 | [-0.77 | -0.07] | **.026** | -0.32 | [-0.67 | 0.14] | .165 | 0.23 | [-0.24 | 0.61] | .327 | 0.15 | [-0.31 | 0.56] | .514 | 0.09 | [-0.37 | 0.51] | .719 | -0.54 | [-0.79 | -0.12] | **.015** |
| 13EI L. Insula/IFG (-38, 20, -6) | -0.08 | [-0.51 | 0.37] | .731 | 0.03 | [-0.42 | 0.46] | .907 | 0.22 | [-0.25 | 0.60] | .352 | -0.30 | [-0.65 | 0.17] | .205 | -0.26 | [-0.63 | 0.21] | .277 | -0.29 | [-0.65 | 0.17] | .212 |
| 14EI L. Parietal (-62, -44, 34) | -0.24 | [-0.62 | 0.23] | .309 | -0.19 | [-0.58 | 0.28] | .433 | 0.05 | [-0.40 | 0.48] | .828 | 0.12 | [-0.34 | 0.54] | .600 | 0.16 | [-0.30 | 0.57] | .490 | -0.25 | [-0.62 | 0.22] | .295 |
| 15EI L. Striatum (-12, 10, -10) | -0.17 | [-0.57 | 0.30] | .479 | -0.07 | [-0.49 | 0.39] | .784 | 0.15 | [-0.32 | 0.55] | .539 | 0.08 | [-0.37 | 0.51] | .734 | -0.12 | [-0.53 | 0.34] | .627 | -0.24 | [-0.62 | 0.22] | .304 |
| 16EI R. Insula/IFG (42, 18, -6) | -0.22 | [-0.60 | 0.25] | .362 | -0.11 | [-0.53 | 0.35] | .633 | 0.21 | [-0.25 | 0.60] | .366 | -0.12 | [-0.54 | 0.34] | .609 | -0.11 | [-0.53 | 0.35] | .636 | -0.31 | [-0.66 | 0.16] | .186 |
| 17EI R. Parietal (58, -44, 30) | -0.33 | [-0.67 | 0.14] | .160 | -0.22 | [-0.61 | 0.24] | .340 | 0.13 | [-0.33 | 0.54] | .584 | -0.08 | [-0.51 | 0.37] | .729 | -0.03 | [-0.46 | 0.42] | .910 | -0.38 | [-0.70 | 0.07] | .098 |
| 18EI R. Striatum (14, 10, -10) | -0.58 | [-0.81 | -0.18] | **.008** | -0.51 | [-0.78 | -0.09] | **.021** | 0.00 | [-0.44 | 0.44] | .990 | 0.29 | [-0.17 | 0.65] | .207 | 0.10 | [-0.36 | 0.52] | .668 | -0.53 | [-0.79 | -0.12] | **.016** |
| 19EI R. pre-SMA (4, 30, 54) | -0.27 | [-0.64 | 0.20] | .250 | -0.28 | [-0.64 | 0.19] | .236 | -0.10 | [-0.52 | 0.35] | .661 | 0.21 | [-0.26 | 0.60] | .373 | 0.26 | [-0.20 | 0.63] | .262 | -0.24 | [-0.62 | 0.22] | .301 |

## **Supplementary Figures**


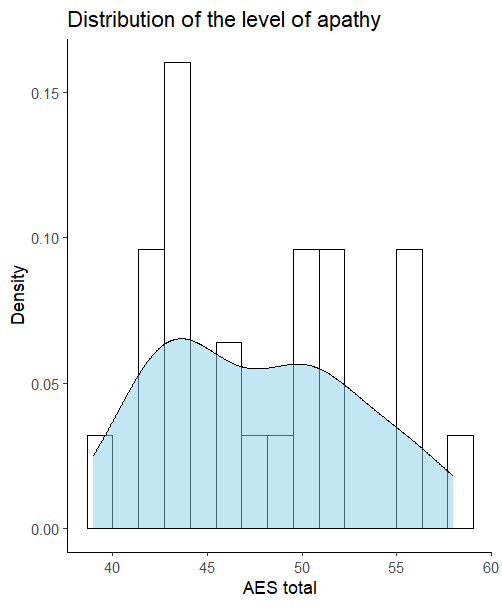


### Figure S1 Distribution of levels of apathy as measured by AES total score. The participants were selected based on a cutoff score of AES-apathy subscale of 27 indicating clinical apathy.


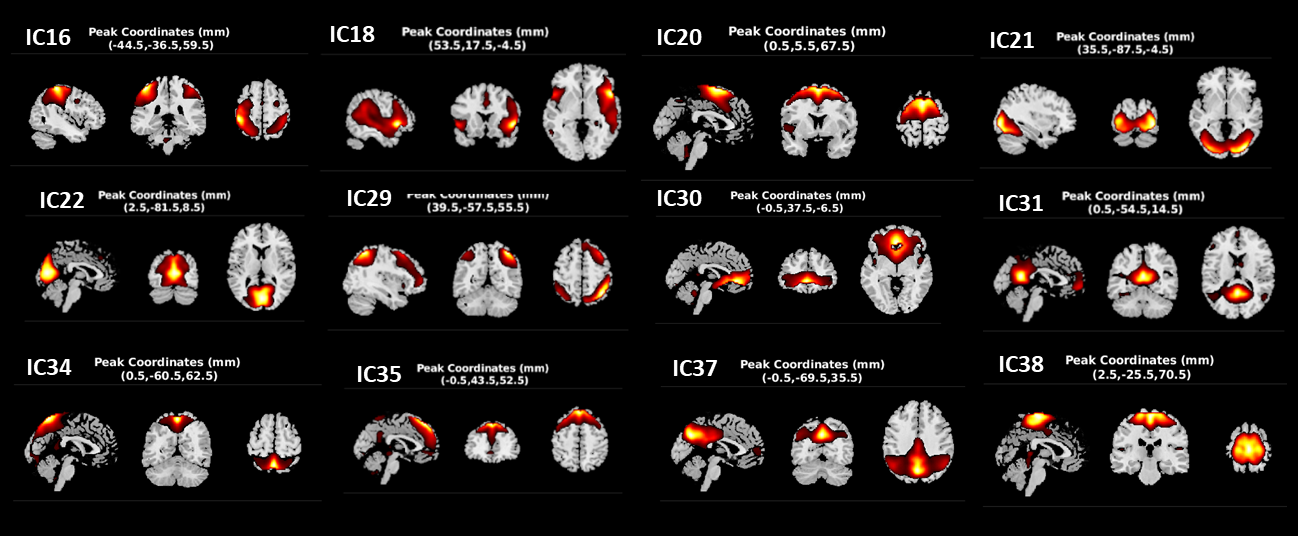


### Figure S2 16 Networks identified through visual selection on the spatial map and spectral power, after excluding 24 ICs reflects artifacts.
